# Investigation of a fully mechanistic physiologically based pharmacokinetics model of absorption to support predictions of milk concentrations in breastfeeding women and the exposure of infants: A case study for albendazole

**DOI:** 10.1002/psp4.13260

**Published:** 2024-11-19

**Authors:** Susan Cole, Maria Malamatari, Andrew Butler, Mahnoor Arshad, Essam Kerwash

**Affiliations:** ^1^ Medicines and Healthcare Products Regulatory Agency London UK; ^2^ University College London London UK

## Abstract

Due to limited non‐clinical and clinical data, European guidance recommends to discontinue breastfeeding when taking albendazole. The aim of this study was to consider the use of PBPK modeling to support the expected exposure in breastfed infants. A fully mechanistic PBPK approach was used to provide quantitative predictions of albendazole and its main active metabolite, albendazole sulfoxide, concentrations in plasma and breast milk of lactating women. The model predicted the exposure in adults and the large food effect, however, it does not predict all the clinical data for the exposure in children. Milk/plasma ratio predictions were also largely over‐predicted for this lipophilic compound, but not for the less lipophilic metabolite. Predictions using the observed ratio and a worse‐case exposure based on *C*_max_ predictions, suggest doses to children through milk will be low. However, more clinical data are required before full exposure predictions can be made to breastfed infants.

**Study Highlights**

**WHAT IS THE CURRENT KNOWLEDGE ON THIS TOPIC?**

Physiologically based pharmacokinetic (PBPK) models are under investigation to predict exposure in breast milk and breast‐fed infants. There are currently few published examples of the use of these models.

**WHAT QUESTION DID THIS STUDY ADDRESS?**

Can PBPK models be used to support the exposure of a low solubility/low bioavailability drug, albendazole, and its metabolite in breast milk?

**WHAT DOES THIS STUDY ADD TO OUR KNOWLEDGE?**

The model captures the exposure in adults with and without food but is less able to capture the exposure of the limited pedatric data available. Milk/plasma ratios predict well for the metabolite but not the parent drug which is highly lipophilic.

**HOW MIGHT THIS CHANGE DRUG DISCOVERY, DEVELOPMENT, AND/OR THERAPEUTICS?**

This example demonstrates that additional clinical data are required to understand the pharmacokinetics of albendazole in children and that improved models are required for milk/plasma portioning.

## INTRODUCTION

Breastfeeding has significant advantages for the mother and baby, especially in the first 6 months.^1^ Many medicines pose varying degrees of harm to a breastfed baby,^2^ therefore use of medicines during breastfeeding is generally not recommended unless the benefits outweigh the risks. Breastfeeding women may reduce the period of breastfeeding if they are uncertain about the adverse effects^3^ of medication on their infant or may avoid taking medicines and prioritize breastfeeding to the detriment of their own health which may be unnecessary for some medicines. This dilemma is particularly problematic for essential medicines including those used to treat parasitic infections, HIV, or post‐partum depression.

Albendazole (ALB) is an anthelmintic agent and a benzimidazole derivative. The mechanism of action of benzimidazoles involves binding to beta‐tubulin in parasitic worms, which results in worm immobilization and eventual demise. Albendazole is indicated in a number of parasitic infections such as Ascaris lumbricoïdes, Ankylostoma duodenale, and strongyloidiasis.^4^ It is contraindicated in pregnant women due to teratogenic effects seen in non‐clinical studies. Some guidance recommends discontinuing breastfeeding when taking albendazole.^5^, ^6^, ^7^ The WHO Model List of Essential Medicines (23rd list—2023)^8^ includes albendazole in section 5, indicating that albendazole is used in a number of mass anthelminthic drug programs to eliminate lymphatic filariasis as a single agent or in combination with either diethylcarbamazine citrate (DEC) or ivermectin.^9^ In filariasis programs, it may be regarded as necessary to give albendazole to lactating women to reach a satisfactory level of worm eradication. Albendazole is given as a single administration or as 400 mg twice a day for 3 days, no time dependency in pharmacokinetics is evident.

The pharmacokinetic (PK) data of albendazole and its main active metabolite, albendazole sulfoxide (ALB‐SO), in lactating women are insufficient.^10^ There has only been one PK study in 33 lactating women who were breastfeeding infants between 2 weeks and 6 months of birth and were administered a single oral dose of 400 mg of albendazole. This was only following a single dose, containing limited milk concentration time points in each subject and only a single serum time point. It was estimated that a breastfed infant would be subjected to less than 0.1 mg/kg of albendazole sulfoxide across a 36‐h duration after a 400 mg maternal dose and even less of albendazole.^11^ The safety of exposing younger children to albendazole is questioned although albendazole has been recommended for children as young as 12 months.^12^, ^13^ The safety profile of albendazole has not been investigated in lactating women and breastfeeding infants 0–12 months old. Regulatory authorities have recently proposed a framework for clinical lactation studies.^14^ However, there are still several logistical and ethical obstacles to learning more about drug exposure in breast milk.^15^

For healthcare professionals and patients to make informed decisions about the benefit/risk balance of using medicines during breastfeeding, quantitative PK data are needed to determine the amount of the drugs distributed in milk along with information regarding infant drug absorption to be able to evaluate the extent of exposure in the infant. The information is often not available as clinical studies are not routinely performed in these populations but performing analyses using a modeling approach could support the considerations. Physiologically based pharmacokinetics (PBPK) modeling has been used to predict clinical PK results in the presence of intrinsic factor(s), such as age, organ impairment, or pregnancy, or extrinsic factor(s), such as formulation, diet, and concomitant medicines.^16^ Gaps in knowledge currently limit the confidence in the models in some populations, for example, very young/newborn children and pregnant women,^17^, ^18^ and regulatory authorities often recommended its use in conjunction with sparse confirmatory PK data.

PBPK also offers an opportunity to predict the concentrations of distributed drugs and metabolites in breast milk and how this can change with time post‐birth. Moreover, PBPK modeling allows the prediction of infant plasma concentrations of the distributed drugs after milk ingestion, accounting also for developmental factors. The predicted concentrations could be used, again with sparse PK data, to better support the benefit/risk decisions and support physicians in making informed decisions on drug dosing in lactating women during breastfeeding. PBPK models that include advanced oral absorption models (ADAM and ACAT) to allow fully mechanistic absorption are considered important to support extrapolation across populations, particularly for low‐solubility drugs. These absorption models are not always included, however, often being replaced with simple first‐order models, in some part this is due to the availability of data and complexity of models.^18^

The aim of this study was to consider the use of PBPK modeling to support the expected exposure of albendazole in breastfeeding infants following a maternal dose of 400 mg of albendazole. This involved the use of a fully mechanistic PBPK approach to provide quantitative predictions of albendazole and its main metabolite, albendazole sulfoxide, concentrations in plasma and breast milk of lactating women. Using these concentrations, the daily dose of albendazole, and its metabolite, that the infant would consume via breast milk could be calculated. The model could also be used to consider the levels of infant exposure to albendazole.

## METHOD

The clinical plasma concentration data in adults and children were collected from the literature for model development and evaluation. The software GetData Graph Digitizer (http://getdata‐graphdigitizer.com/) was used to digitalize the data from the graphs available in the literature.

A model was developed de novo using the software platform SIMCYP Population‐Based Simulator (Version 23 Release 1; SIMCYP Limited), based on collected in vitro and in vivo input data publicly available. The majority of the parameters were sourced from the publications by Pettarin et al.^19^ and Shah et al.^20^ which contain a detailed description of the in vitro biopharmaceutical characterization and absorption characteristics. The input parameters for the model are shown in Table 1.

**TABLE 1:** Input parameters for the albendazole and albendazole sulfoxide PBPK model.

Parameter	Value	Source
Albendazole		
Molecular weight	265.34	
Log *P*	3.46	21
pKa	10.26, 2.8	22
Drug type	Ampholyte	
Blood/Plasma	0.74	19
PPB (Fu)	0.09	23
Absorption		
Model	ADAM with unstirred boundary layer	
Papps ×10^−4^ cm s^−1^	1.835	19
Solubility @pH 6.8 μg/mL	2.0	19
Bile micelle partition coefficients	5.5	Optimized
Formulation	Solid immediate release	
Supersaturation ratio	2.86	19
Precipitation rate (h^−1^)	First‐order immediate 6.46	19
Particle size (μm)	Polydisperse Diffusion layer model Mean = 8.6817 Weibull alpha = 1.014, beta = 8.732	20
Gut vols	Stomach‐ 50 mL Jejunum to ileum 105 mL Colon 13 mL	24
Distribution		
Method	Full PBPK	
Kps method	Rogers and Rowland	
*V*_d_ (L/kg)	11.2	
Milk/plasma	0.1	Predicted
Clearance		
CYP3A4 *V*_max_ pmol/min/mg	2200	Optimized based on^25^
Km μM	4	
FMO3 *V*_max_ pmol/min/mg	768	Optimized
Km μM	3.59	
Microsome binding fu	0.89	
Albendazole sulfoxide		
Molecular weight	281.34	
Log *P*	1.15	20
pKa	9.79, 0.2	20
Drug type	Ampholyte	
Blood/Plasma	0.77	19
PPB (Fu)	0.3	19
Distribution		
Method	Full PBPK	
Kps method	Rodgers and Rowland, Kpscalar = 3	Optimized
*V*_d_ (L/kg)	1.0	
Milk/plasma	0.3	Predicted
Clearance		
CYP3A4 Clint μL/min/рmol	0.0823	Retrograde based on DDI
Microsome binding Fu	1.0	
Clrenal (L/h)	0.49	Clinical data

A fully mechanistic oral absorption was used for all the simulations using the ADAM model for absorption incorporating the unstirred boundary layer (UBL) and with measured dissolution and precipitation.

Albendazole is an ampholyte with a basic pKa of 2.80 and an acidic pKa of 10.26.^22^ Albendazole exhibits desmotropism, a phenomenon related to tautomerism, in which both forms can be isolated in the solid state.^26^ Pettarin et al.^19^ characterized the two solid forms of albendazole, form I and form II using thermal analysis, diffraction studies, and solubility experiments. Characterization of the commercial product 400 mg tablets showed that the formulation consists mainly of form I.^19^ Thus, simulations were performed with albendazole being entirely in form I.

Albendazole has low to moderately permeable drug with its permeability being driven by passive diffusion.^22^ Based on rat jejunal permeability values,^27^ the human permeability of albendazole using GastroPlus v9.7 was estimated to be 1.835 × 10^−4^ cm/s.^19^

Albendazole exhibits pH‐dependent solubility, with solubility greater in gastric environment which drops significantly in the intestinal environment.^28^ Pettarin et al.^19^ determined the solubility of albendazole's form I in biorelevant media. The solubility of albendazole used in the simulations was the solubility of the drug in FaSSIF‐V1/2 mix prepared in a ratio of 1:1.4, which was found to be 2.03 ± 0.56 μg/mL.

The pH‐dependent solubility of albendazole suggests supersaturation and precipitation when the drug moves from the stomach to the small intestine. Based on in vitro data using a combination of turbidity and supernatant microplate assay, albendazole is expected to precipitate rapidly upon gastric emptying.^29^ The low solubility of albendazole along with its precipitation in the small intestine have been highlighted as the main reasons for low oral systemic exposure to albendazole sulfoxide.^30^ Transfer experiments from a FaSSGF (gastric) compartment to a FaSSIF (intestinal) compartment confirmed the supersaturation and precipitation characteristics of albendazole.^19^, ^20^ Specifically, in transfer experiments for the Eskazole® 400 mg tablets, the critical supersaturation ratio (which is determined as the ratio of the critical supersaturation concentration to equilibrium solubility) was determined to be 2.86, while the precipitation rate constant was 6.46 h^−1^.^19^

Particle size distribution data of the drug substance using laser diffraction were reported by Shah et al.^20^ Particle size distribution was characterized as a Weibull function.^31^ Weibull parameters alpha and beta were used as the input parameters in the model development. In order to fully characterize the relatively rapid dissolution of albendazole it was noted important to include additional components to the solubility with the use of the UBL in the ADAM model and high micelle partitioning.

Distribution was described by a full PBPK model with Kps calculated using the equations of Rogers and Rowland, a scaling factor of 3 was also required for the Kps for the sulfoxide metabolite to capture the elimination phase. Clearance values were based on in vitro determination of metabolic pathways by Rawden, these were used with the results of drug interaction studies with P450 inhibitors to define the relative contribution of CYP4A4 and FMO3, however, a scaling factor of 6 was required to adequately capture the in vivo clearance. These enzymes were incorporated in the full PBPK model with *V*_max_ and Kms for the main clearance pathways.

Model building and evaluation steps for the proposed application are outlined below:

1. The drug model was built and initially evaluated in healthy volunteers (HVs) in the fasted state, for which plasma concentration data was available for the parent and metabolite. Evaluation of the adult model involved comparing the simulated plasma concentration–time profiles with those published in the literature. Unless otherwise stated, simulation follows a study design of 10 trials with 10 subjects per trial. Simulated mean and 5th and 95th percentile intervals were overlaid with reported data to investigate model prediction and visually compare profiles. In addition to the comparison of the simulated concentration–time profiles with the clinical pharmacokinetic (PK) data, simulated and published areas under the plasma concentration–time curve (AUC), maximum plasma concentration (*C*_max_) were compared. A twofold error range is generally considered to be the maximum range for a reasonable prediction.
2. The model was verified using data in the fed state and with consideration of known drug interactions.
3. The model was then combined with the pediatric physiology model in SIMCYP to simulate the drug PK in children. This model describes the pediatric population based on pediatric demography (age, height, weight, and body surface area), developmental physiology (liver size, renal function, and liver blood flow), and biochemistry, including CYP enzyme ontogeny, the underlying equations describing these changes have been extensively described elsewhere.^32^ Demographic characteristics (e.g., age) were matched with the data reported in the published clinical studies. The simulated exposures were compared with observed data as for adults.
4. Simulations were performed in breastfeeding women 18–45 years including the lactation module in SIMCYP. This model includes the breast as an additional compartment in the model and has also been described elsewhere.^2^ The current model uses the ‘phase distribution’ model to predict the milk‐to‐plasma ratio.^33^, ^34^ The milk concentrations were predicted and compared with collected literature data. Based on the milk concentration data, an infant daily dose (IFD) was calculated.
5. IFD were used to simulate exposure in infants following maximal breastfeeding for worse‐case scenarios.

### Prediction of milk‐to‐plasma ratios

M/P ratios were predicted using the phase distribution mode^15^, ^33^, ^34^

(1) $$M/P\;\text{ratio}=\frac{{\mathrm{fu}}_{p}\cdot f_{p}^{\mathrm{un}}}{{\mathrm{fu}}_{m}\cdot f_{m}^{\mathrm{un}}\cdot S/W\;\text{ratio}}$$

where *fu* and *f* ^un^ are the fraction unbound and fraction unionized respectively, in the plasma (*p*) and milk (*m*). The S/W (skim‐to‐whole milk) ratio represents drug accumulation within the lipid phase of the milk and is calculated as:

(2) $$S/\text{Wratio}=\frac{1}{1+\mathrm{Crt}\cdot \left({\mathrm{fu}}_{m}\cdot P_{\text{milk}}-1\right)}$$

where the Crt is the creamatocrit and was set at 3.9% for all simulations. *P*_milk_ is the partition coefficient between the aqueous and lipid phase of milk, and was calculated as^35^:

(3) $$P_{\text{milk}}=10^{-0.88+1.29\text{LogD}7.2}$$

Ionized fraction was calculated using Henderson‐Hasselbalch equations and a milk pH of 7.0. Fraction unbound in the milk was estimated as previously reported using *fu*_*m*_ ^36^:

(4) $${\mathrm{fu}}_{m}=\frac{{{\mathrm{fu}}_{p}}^{0.448}}{{\left(6.94\times 10^{-4}\right)}^{0.448}+{{\mathrm{fu}}_{p}}^{0.448}}$$

### Calculation of relative infant daily dose

Two methods (*C*_ave_ and *C*_max_) were employed to calculate the relative infant daily dose (RIDD) to represent average exposure and a worst‐case scenario. In both methods:

$$\text{RIDD}\;\left(\%\right)=\frac{\text{Infant daily dose}\;\left(\mathrm{mg}/\mathrm{kg}/\mathrm{day}\right)}{\text{Maternal daily dose}\;\left(\mathrm{mg}/\mathrm{kg}/\mathrm{day}\right)\;}\times 100$$

In the *C*_ave_ method, the infant daily dose was calculated using the average predicted concentration of drug within the milk, while the *C*_max_ method used the maximum milk concentration. Both methods assumed infant consumption to be 150 mL/kg/day.^37^, ^38^

## RESULTS

### Evaluation of the model in adults

A number of studies report the PK of albendazole and its metabolites in adults. In many cases, however, there are only data for the main active metabolite, the sulfoxide, which is evident in concentrations significantly higher than that of the parent. To fully evaluate the model, only studies with clinical data for both parent and metabolite were used and initial model development was based on data in the fasted state. This state should be more sensitive to the low solubility which limits the absorption of albendazole. Model parameters were initially selected by comparison of predicted profiles with the data from Corti et al.^39^

**FIGURE 1:**
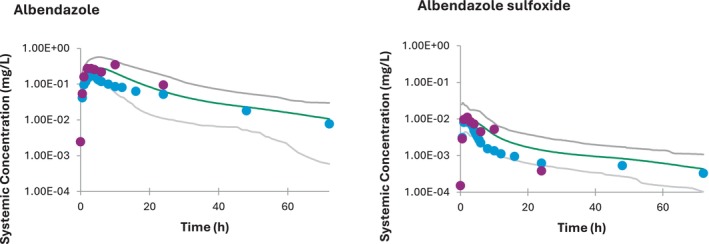
Systemic concentration of albendazole (left) and albendazole sulfoxide (right) in adults following a single 400 mg oral dose of albendazole. Solid lines represent the mean predicted values and dashed lines represent the 95% confidence intervals. Blue dots represent the data observed by Corti et al., and yellow dots represent the data observed by Rathod et al.

Simulations show a reasonable fit to the data for the parent and sulfoxide metabolite (Figure 1) and PK parameters showed a reasonable agreement with an observed *C*_max_ of 10.1 and 330 ng/mL compared to the predicted ones of 15.4 and 453 ng/mL for albendazole and albendazole sulfoxide, respectively. Observed AUC0‐24h were 135 (ALB) and 3440 mg/L h (ALB‐SO), compared to the predicted values of 105 and 4110 mg/L h for albendazole and the sulfoxide, respectively. The terminal elimination, however, was not well characterized in this clinical study. Additional data in the fasted state with extended time points were available in the study of Rathod et al.^40^ and this was also the one used in the model described by Pettarin et al.^19^ from which the majority of the model parameters were taken. It is noted that the exposure of both parent and metabolite is lower in this study. Verification of the model by comparisons with the predicted profiles showed an over‐prediction (Table 2), however, the elimination phase appears to be reasonably well characterized (Figure 1). Thus, all available data in the fasted state were used in model development and there were insufficient data available to allow an independent test of the model.

**TABLE 2:** Predicted versus observed pharmacokinetic parameters in adult healthy volunteers.

Simulation	Observed^27^	Observed^28^	Predicted	Predicted	Observed^29^
Dose (mg)	400	400	400	400	400
Fed state	Fasted	Fasted	Fasted	Fed	Fed
ALB AUC (μg h/L)	100.1 ± 110.8 (0–24 h)	53.4 ± 36.00 (0–72 h)	103 (0–24 h) 135 60–300 (0–72 h)	670 310–1960	642.9 ± 762.8
ALB *C*_max_ (μg/L)	15.3 ± 10.3	11.5 ± 4.4	10.1 0–30	73 40–240	128.9 ± 160.4
ALB‐SO AUC (μgh/L)	5441.3 ± 4725.2 (0‐24 h)	3306.3 ± 1741.5 (0–72 h)	4010 (0–24 h) 4900 2020–11,370 (0–72 h)	18,200 9990–48,620	19,919.9 ± 16,139.5
ALB‐SO *C*_max_ (μg/L)	453.9 ± 398.7	207.06 ± 59.55	330 150–570	1440 900–2550	1580.2 ± 1080.2

*Note*: Predicted data shows median plus 5‐ 95th percentiles, observed data are reported as mean ± SD.

Verification of the model was performed by running simulations in the fed state, which is expected to increase exposure significantly.^41^ In support of the model reliability, the fed state led to a 4.96‐ and 3.71‐fold increase in AUC of ALB and ALB‐SO, respectively (Table 2). These values were similar to those observed by Ochoa et al.^41^

### Evaluation of the model in children

There are very limited data on children with only three studies being identified.^23^, ^42^, ^43^ These were generally in older children. Simulations were run in 11–17 year olds, ratio 3:10 female to male to match the data from Pengsaa et al.,^42^ which contained the data in the biggest number of subjects (*n* = 10). It is difficult to match the fed state of simulations exactly to that of this study, where ALB was administered “immediately after each child had drunk 200 ml ultra‐heat treatment (UHT) milk, approximately 1 hour after breakfast”. When simulations were run in a fed state, a large over‐prediction was seen (P/O ratio for AUC = 4.28), but in the fasted state the predicted exposure was similar to those observed (P/O ratio for AUC = 1.53; Table 2).

Clinical data in young children are very limited. There are only data in two children (1 male: 1 female) less than 2 years of age, (1.9 years^23^), predictions in fasted state alone show a large over‐prediction (Table 3).

**TABLE 3:** Predicted versus observed albendazole PK parameters in children.

	Observed		Predicted		
Age (years)	11–17	1.9	11–17	11–17	1.9
Dose	200 mg	15 mg/kg	200 mg	200 mg	15 mg/kg
Fed/Fasted	200 mL UHT milk, 1 h after breakfast *n* = 10	Suspension with olive oil *n* = 2	Fed	Fasted	Fasted
ALB AUC (μg h/L)	ND		834 350–1470	214 80–380	727 350–1880
ALB *C*_max_ (μg/L)	45.5 (0–172)	<50	104 50–190	17.6 10–40	112 40–290
ALB‐SO AUC (μg h/L)	4952 (2257–14,138)	1700–2800	21,200 870–42,410	7610 2850–13,870	22,800 10,730–58,6000
ALB‐SO *C*_max_ (μg/L)	952 (545–2000)	400–600	2140 930–2740	645 280–840	1970 920–3030

*Note*: Data presented as median and range for observed data or median and 95th percentiles for predicted.

### Evaluation of predicted milk concentrations

There is only one study which reports milk concentrations in women who are breastfeeding.^11^ Milk concentrations were measured following a 400 mg dose given with breakfast to breastfeeding women (*n* = 33). Milk concentrations were analyzed for the parent drug and the metabolite. There was only one serum sample taken at 6 h.

The predicted ratio milk/plasma (M/P) ratio was 13.5 and 0.36 for albendazole and albendazole sulfoxide, respectively. The value for albendazole is much higher than the observed value reported in the publication of 0.9, while the sulfoxide value is only slightly lower than that reported of 0.6. Adjusting the creamatocrit to capture the physiological variation in breast milk (3–12%) did not improve predictions for the parent (M/P range = 10.4–41.4) or significantly alter the predictions for the metabolite (0.35–0.42), and both compounds were insensitive to changes in milk pH.

Predictions for breastfeeding women (Table 4) showed an over‐prediction of the serum concentration at 6 h for the metabolite. Predictions using the observed milk/plasma ratio showed reasonable predictions for the parent albendazole but with a moderate over‐prediction ~2‐fold for the sulfoxide. Using predicted M/P ratios resulted in a large over‐prediction of the parent, but was closer for the metabolite.

**TABLE 4:** Observed versus predicted serum and milk concentrations in breastfeeding women.

Compound	Observed concentration	Predicted (Pred M/P ratio)	Predicted (Obs M/P ratio)	Observed concentration	Predicted (Pred M/P ratio)	Predicted (Obs M/P ratio)
	Albendazole			Albendazole SO		
Serum 6 h	63.7 ± 11.9	39.8^a^ 13.7–92.8^a^	39.8^a^ 13.7–92.8	608.0 ± 10.7	1430^a^ 604–2430^a^	1430^a^ 604–2430
Milk 6 h	31.9 ± 9.2	621 164–1480	35.8 12.4–83.4	312.8 ± 30.6	525 226–939	857 362–1460
Milk 12 h	18.8 ± 6.7	228 59.1–572	13.4 0.36–29.5	225.7 ± 27.7	285 73.6–569	467 110–917
Milk 24 h	7.5 ± 2.9	101 18.9–271	6.0 1.6–13.7	94.1 ± 7.0	85.9 8.83–222	141 12.7–377
Milk 36 h	ND	59.9 13.2–133	3.6 1.0–6.7	57.1 ± 11.1	35.1 4.48–103	57.7 7.1–173

*Note*: Predicted albendazole and albendazole sulfoxide concentrations in the milk were generated using the lactation model presented in Equations (1), (2), (3), (4) (Pred M/P ratio) and using the clinically observed data (Obs M/P ratio).

^a^ Serum concentrations were predicted independently of the M/P method utilized, thus are the same in each predicted column. Predicted data shown as medians and 95th percentiles and observed values as mean ± SE.

The observed M/P ratios were used to predict the expected doses of albendazole and its sulfoxide in breastfed infants using the expected milk consumption of 150 mL/kg/day given over six doses (every 4 h) and based on (a) predicted *C*_ave_ and (b) predicted *C*_max_ concentrations in milk. The results are shown in Table 5.

**TABLE 5:** Predicted infant exposure and daily dose.

	AUC_0‐72_ (mg/L h)	*T*_max_ (h)	*C*_max_ (mg/mL)	*C*_ave_ (mg/mL)	Infant daily dose (mg/kg/day)		Relative infant daily dose (%)	
					*C*_ave_ method	*C*_max_ method	*C*_ave_ method	*C*_max_ method
Albendazole	0.064	1.15	75.9 80	38 40	0.006	0.011	0.29	0.58
Albendazole sulfoxide	11.900	4.55	978 910	489 450	na	na	na	na

Based on a worst‐case prediction, where albendazole and albendazole sulfoxide are consistently at *C*_max_ during each feed, the calculated infant daily dose is 0.6% of the adult dose for albendazole equating to doses of 0.011 mg/kg/day. It is difficult to calculate a relative infant dose of the metabolite as this is a metabolite and thus not dosed directly. Additional metabolite will be produced following its administration in milk with the further metabolism of albendazole in the infant.

### Predicted infant exposure

**FIGURE 2:**
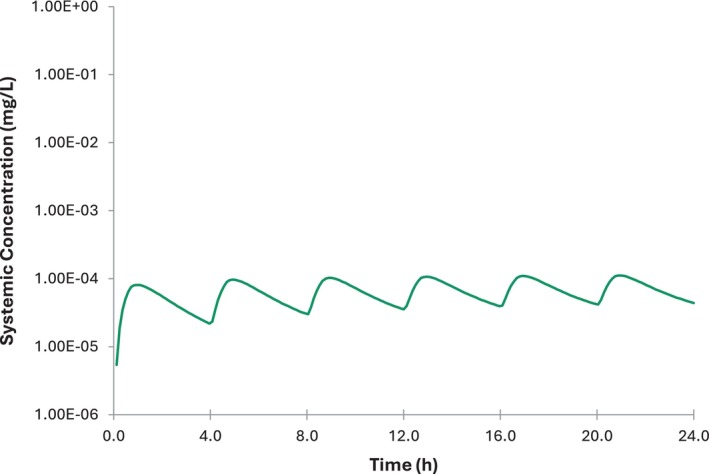
Predicted systemic concentration of albendazole. The dosing regimen was based on a 4‐hourly pattern of breastfeeding. The *C*_max_ of ALB and the observed M/P ratio was used to estimate dosage. Solid lines represent the mean predicted values.

Simulations performed for breastfed infants using the observed ratios and infant daily dose based on *C*_max_ concentrations are shown in Figure 2. These simulations assumed breastfeeding every 4 h, therefore, the daily dose divided by 6 and assumed to be taken with food as the worst case. The age of the infant population used was 0–12 months.

Some accumulation is seen but this is moderate, ~2‐fold. Plasma concentrations for albendazole are at least 100‐fold lower than those seen in adults at the recommended clinical dose.

## DISCUSSION

We have developed a fully mechanistic absorption model of the low solubility drug albendazole which incorporates solubility, dissolution, supersaturation, and precipitation. The model is very sensitive to the solubility data and gut volumes used in the model. The main clearance pathways were also included mechanistically in the model.

The initial model development was based on the clinical data from the paper Corti et al. which investigated the PK following dosing of 400 mg to healthy volunteers in the fasted state and were the data used in the model by Shah et al.^20^ It should be noted that this paper investigated the effect of the CYP3A4 inhibitor, ritonavir. However, the data used were from the control arm without ritonavir. The data described by Rathod et al.,^40^ despite using a similar population of HVs show lower exposure. The reason for this difference is not obvious, the second population was of Indian race and could have included some females, however, these factors would be expected to have a lower body weight, so may be expected to have the opposite effect, with higher exposure. The difference between studies may simply reflect the expected variability for a low‐solubility drug between clinical studies. A reasonable fit, however, was seen for both studies with some over‐prediction which will give the worst‐case scenario in the application to breastfeeding. The absorption profile appeared to be reasonably captured by the model suggesting absorption had been well characterized in adults.

A number of studies performed following dosing with food showed a large increase in exposure which would be expected for a low‐solubility drug.^41^, ^44^, ^45^ It is considered important that the effect of food be well characterized for this application as predictions in breastfed infants will involve administration with some food, for example, milk. Simulations in adults showed good agreement with the observed fed data. The observation of the effect of P450 inhibitors, for example, grapefruit juice^41^ also agreed with model parameterization and simulations (data not shown).

There are very limited data on children and all studies were performed in affected children.^23^, ^42^, ^43^ In all these studies attempts were made to administer with food to increase the exposure, for example, Pengsaa et al.^42^ gave the drug with milk and 1 h. after breakfast while Jung et al.^22^ dosed with olive oil. However, the exposure is low compared to predictions particularly in the younger children (1.9 years). It is noted that data are very limited (*n* = 2) and unexpectedly low, in these two subjects, considering the dose given. The authors noted the low exposure, but no explanation was provided. Interestingly, the observed exposure seems more similar to that predicted in the fasted state, suggesting the food effect may be less, or even absent in children. In agreement with this finding, in the Ochoa et al.^41^ study, children were studied following dosing in the fasted state, and after a high‐fat breakfast, and no difference in exposure was observed. The exposure was also low in all individuals. The model does predict a smaller food effect in children, however, it is still predicted in 2‐year‐olds to increase exposure 2.7‐fold compared to 3.7‐fold in adults. The reason for the low exposure in children and apparent lack of food effect is not known. It could represent lower absorption not currently predicted by the model, higher clearance in children due to unique or an increase in existing pathways, or reliability of the clinical data due to factors in the clinical study. Given the low subject numbers, additional clinical data in well‐designed studies would be required to confirm these findings. This limits confidence in the model for predictions in young children, however, it can be expected that exposure will be over‐predicted.

The predictions for women in the Abdel‐tawab et al.^11^ study also showed an over‐prediction of serum concentrations for the metabolite, although only one time point was taken. It is assumed that serum and plasma concentrations are equivalent. This over‐prediction could also be due to an overestimation of the food effect, as the exact content of the breakfast used in this study is not known. Milk concentrations of albendazole sulfoxide also are over‐predicted when calculated using the observed M/P ratio. However, this is primarily due to the over‐prediction of serum concentration. The predicted M/P ratio is greatly over‐predicted for albendazole but not for the sulfoxide. Such over‐prediction using the phase distribution model has been noted for other lipophilic compounds and improved methods are being developed.^46^ With this in mind, it should be noted that the equation derived by Atkinson and Begg for the prediction of lipid partitioning in the milk (Equation 3) was based on compounds with a LogD_7.2_ of between −0.5 and 3. Linear extrapolation of this dataset is (perhaps incorrectly) assumed. The LogD_7.2_ of albendazole and albendazole sulfoxide was calculated to be 3.46 and 1.57 respectively, which may go some way to explaining the over‐prediction of ALB concentration within the milk. Additionally, the reliability of the observed M/P ratio may be limited by the collection of paired plasma samples at a single time point only (6 h). The predicted M/P ratios represent those in the steady state, and so are better suited to be compared with AUC M/P ratios than single time point measurements. Given that M/P ratios can change over time post‐dose, additional clinical studies with which more detailed PK profiling would be beneficial and further support model validation.

Using the observed milk ratio in the model, and *C*_max_ values, as a worse case, this equates to doses of 0.014 and 0.147 mg/kg/day for albendazole and albendazole sulfoxide, which is 0.6% of the adult dose for albendazole, this is within the recommended less than the 10%.^47^, ^48^

The approach used here has been used to predict exposure in breastfed infants and to understand exposure in milk at different times post‐partum.^49^, ^50^ There are limitations and assumptions in the PBPK models for children and lactation as these are built on the data that are available and these are sometimes limited, for example, gut pH in children and ontogeny of transporters and protein binding in milk. In this application, particularly assumptions around the absorption model in infants, the absence of some minor clearance pathways for the drug, as well as uncertainties on factors involved with determining partitioning into breast milk, for example, milk binding will have a large impact. The limited data available for model verification are also an issue. Given the caveats, for this model, in terms of the misprediction of M/P ratio and of exposure in infants, there is uncertainty in the prediction to breastfed infants and, therefore, quantitative conclusions, particularly on systemic exposure in infants, cannot be made with confidence but can be assumed to be conservative. The prediction of the food effect and exposure in children requires further work and a similar modeling approach is being used for other drugs to determine if this is a general finding.

In summary, we have developed a detailed fully mechanistic PBPK model for the low‐solubility drug albendazole. The model predicts the exposure in adults and the marked food effect and reasonably predicts milk concentrations using the observed M/P ratios. Despite the fully mechanistic absorption model, however, it does not predict the available clinical data for exposure and food effects in children. We demonstrate how this model could be used to predict exposure in breastfeeding infants. However, predictions for milk partitioning also show mis‐prediction for the parent drug, limiting the conclusions that can be made from a purely in silico approach.

## AUTHOR CONTRIBUTIONS

S.C., M.M., A.B., and E.K. wrote the manuscript. S.C., M.M., A.B., and E.K. designed the research. S.C., M.M., A.B., and A.H. performed the research. S.C., M.M., A.B., and A.H. analyzed the data.

## FUNDING INFORMATION

This work was funded by the Bill and Melinda Gates Foundation (INV‐009383). The views expressed in this work do not reflect the official views of the Bill & Melinda Gates Foundation.

## CONFLICT OF INTEREST STATEMENT

The authors have no conflicts of interest.
